# Review of Early Immune Response to SARS-CoV-2 Vaccination Among Patients With CKD

**DOI:** 10.1016/j.ekir.2021.06.027

**Published:** 2021-07-06

**Authors:** Edward J. Carr, Andreas Kronbichler, Matthew Graham-Brown, Graham Abra, Christos Argyropoulos, Lorraine Harper, Edgar V. Lerma, Rita S. Suri, Joel Topf, Michelle Willicombe, Swapnil Hiremath

**Affiliations:** 1 The Francis Crick Institute, London, UK; 2 Department of Medicine, University of Cambridge, Cambridge, UK; 3 Department of Cardiovascular Sciences, University of Leicester, Leicester, UK; 4 Satellite Healthcare, San Jose, California, USA; 5 Division of Nephrology, Department of Medicine, Stanford University, Palo Alto, California, USA; 6 Division of Nephrology, Department of Internal Medicine, University of New Mexico School of Medicine, Albuquerque, New Mexico, USA; 7 Institute of Applied Health Research, University of Birmingham, Birmingham, UK; 8 Section of Nephrology, University of Illinois at Chicago/Advocate Christ Medical Center, Oak Lawn, Illinois, USA; 9 Research Institute, University Health Center, Department of Medicine, McGill University, Montreal, Québec, Canada; 10 Department of Medicine, Oakland University William Beaumont School of Medicine, Detroit, Michigan, USA; 11 Department of Inflammation and Immunity, Imperial College, London, UK; 12 Department of Medicine, University of Ottawa, Ottawa, Ontario, Canada

**Keywords:** coronavirus, COVID, COVID-19, dialysis, kidney disease, transplant, vaccine

## Abstract

The effects of the coronavirus disease-2019 (COVID-19) pandemic, particularly among those with chronic kidney disease (CKD), who commonly have defects in humoral and cellular immunity, and the efficacy of vaccinations against severe acute respiratory syndrome coronavirus-2 (SARS-CoV-2) are uncertain.

To inform public health and clinical practice, we synthesized published studies and preprints evaluating surrogate measures of immunity after SARS-CoV-2 vaccination in patients with CKD, including those receiving dialysis or with a kidney transplant.

We found 35 studies (28 published, 7 preprints), with sample sizes ranging from 23 to 1140 participants and follow-up ranging from 1 week to 1 month after vaccination. Seventeen of these studies enrolled a control group. In the 22 studies of patients receiving dialysis, the development of antibodies was observed in 18% to 53% after 1 dose and in 70% to 96% after 2 doses of mRNA vaccine. In the 14 studies of transplant recipients, 3% to 59% mounted detectable humoral or cellular responses after 2 doses of mRNA vaccine. After vaccination, there were a few reported cases of relapse or *de novo* glomerulonephritis, and acute transplant rejection, suggesting a need for ongoing surveillance.

Studies are needed to better evaluate the effectiveness of SARS-CoV-2 vaccination in these populations. Rigorous surveillance is necessary for detection of long-term adverse effects in patients with autoimmune disease and transplant recipients. For transplant recipients and those with suboptimal immune responses, alternate vaccination platforms and strategies should be considered. As additional data arise, the NephJC COVID-19 page will continue to be updated (http://www.nephjc.com/news/covid-vaccine).

## Graphical abstract

**Figure:**
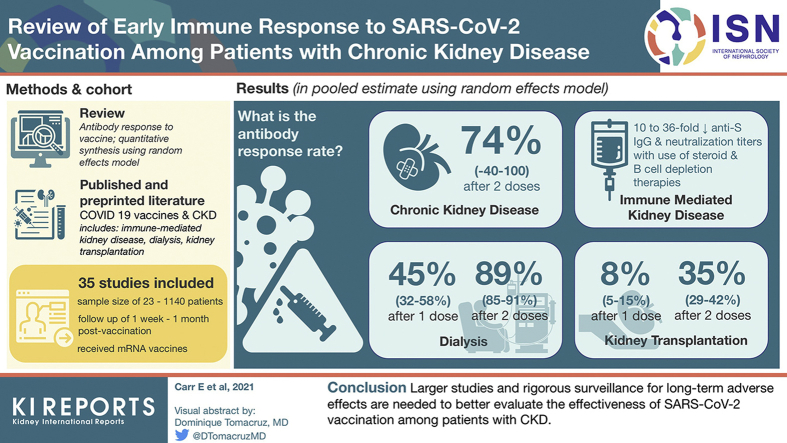

The effects of the COVID-19 pandemic have been far-reaching and have affected people in all parts of the world. Few, if any, patient groups have been affected to the same extent as those with chronic kidney disease (CKD). These patients have among the poorest outcomes if contracting the virus. Patients on unit-based hemodialysis (HD) have been unable to shelter in place to protect themselves.^1^^,^^2^

Early and rapid vaccination is an absolute priority for this at-risk group; however, although the results of vaccine trials have been impressive in the general population, the effectiveness of these vaccines has not been explicitly tested in patients with CKD, who are often excluded from trials (Table 1).^3^ At present, clinical vaccine efficacy, immunogenicity, and persistence of protection are “known-unknowns” in patients with CKD (including transplant recipients) and those with end-stage kidney disease (ESKD).^4^^,^^5^ Understanding the response to vaccination in patients with CKD is a top priority for researchers and health care professionals who treat people with kidney disease. Seroconversion to the hepatitis B vaccine declines with advancing kidney disease and seroconversion after the influenza vaccination is variable in patients on dialysis.^6^ Thus, it is not surprising that the international community has moved so quickly to gather and report data on the immune response to vaccination against COVID-19 in patients with kidney disease.

**Table 1:** Summary of COVID-19 vaccine platforms and status

Platform	Target	Examples	Status	Trials in dialysis or transplant?
mRNA	S protein	BNTb162b2 (Tozinameran/Comirnaty, Pfizer/BioNTech), mRNA-1273 (Moderna), CVnCoV (Curevac) PTX-COVID19-B (Providence)	BNT1262b and mRNA-1273 approved in many jurisdictions; others under development	No
Adenovirus vector	S protein	AZD1222 (Vaxzevria/CoviShield, AstraZeneca/Oxford), Ad26.COV2.S (Janssen), Sputnik V (Gamaleya), Convidecia (CanSino)	Approved in many jurisdictions	No
Inactivated protein subunit	S protein	NVX-CoV2372 (Novavax), Epivac Corona (Vector Institute), GSK/Sanofi candidate vaccine	Approved in some countries	No
Inactivated virion	Whole virus	CoronaVac (Sinovac), Covaxin (Bharat Biotech), CoviVac (Chumakov Centre, Russia)	Approved in some countries	No
Others	Whole virus, S protein	DNA vaccines, live attenuated virion, other viral vectors	Under development	No

Presently, the data available are heterogeneous, in early phases, and have been reported in different ways, which makes it difficult to make direct comparisons and provide quantitative synthesis. Nevertheless, these early data are useful and provide a preliminary overview of the protection vaccination may confer for patients with kidney disease, accepting that none of the studies have reported the effects of vaccination on infection or mortality---but instead the immune response---mostly as antibodies with a few studies on cellular activity.

In this narrative investigation we review and discuss the possible implications of the early vaccination data, while acknowledging this is a rapidly evolving area. Continued rigorous work and collaboration are essential to truly understand the protection vaccination will or will not afford patients with kidney disease, and to inform the current and future vaccination strategies.

## Methods

**Figure 1:**
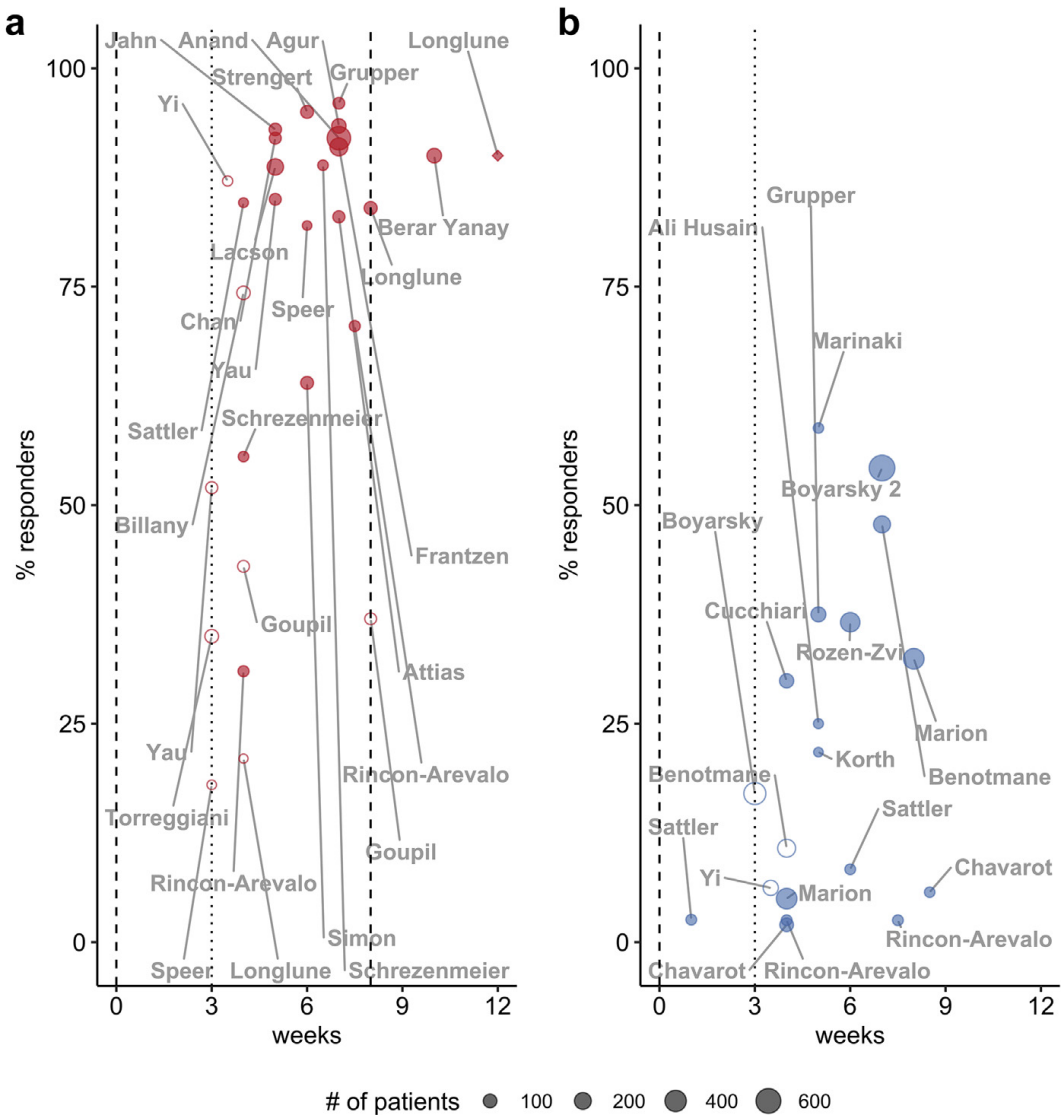
Graphical summary of antibody response over time from all studies. Percentage seroresponse as reported in published or preprinted studies in hemodialysis (a) or renal transplantation (b) patient groups. Data after 1 (open circles), 2 (filled circles), or 3 doses (filled diamond) are shown separately. The size of each point reflects the number of patients tested at that timepoint. Each point is labeled with its first author (full citations are in Tables 2 and 3). Studies used a variety of different measures of antibody responses and, where immunoglobulin isotypes were reported separately, we retained IgG data alone. Where baseline serology is known, we used seronaive vaccine recipient data. For Yi *et al*. and Rincon-Arevalo *et al*., there are both transplant and a smaller number of hemodialysis patients (both subgroups shown).

This is a narrative review of the published and preprinted literature on COVID-19 vaccines in CKD. We performed a literature search, with a hand search of PubMed and all nephrology and transplant journals since the COVID-19 vaccination began. In addition, 2 preprint servers (biorxiv and medrxiv) were searched using the terms “covid,” “vaccine,” and “kidney” or “transplant” or “dialysis.” Bibliographies of the included studies were reviewed for any eligible studies. Last, experts in the field and members of this workgroup reported additional studies the searches had missed. Data from the included studies were extracted by 2 authors (EJC and SH), including details about the patient population, antibody response, number of vaccine doses, type of antibody assay, safety data, and comorbidities. If possible, we removed individuals who were seropositive at the start of the study (i.e., already had a primary infection) and who had incomplete data (e.g., baseline but no postvaccination results), and, again whenever possible, report percentage seropositivity for IgG alone. Our synthesized data are shown in Tables 2 and 3, and the numbers of patients and percentages are plotted against time in Figure 1. The main outcome presented in this review is antibody response. A pooled estimate of the antibody response was performed using the random effects model described by Dersimonian and Laird.^7^

**Table 2:** Vaccine response data in dialysis population

Study	Vaccine	Timing after dose	Sample size^a^	Antibody response	Control group
First-dose studies					
Goupil *et al.*	BNT162b2	4 weeks	131	43%	40 health care workers; Ab levels significantly lower in HD vs. control
Torreggiani *et al.*	BNT162b2	3 weeks	99	37%	None
Billany *et al.*	BNT162b2 or AZD1222	28 days	74	73%	None
Yi *et al.*	BNT162b2 or mRNA-1273	28 days	31	87%	Transplant patients
Attias *et al.*^b^	BNT162b2	28 days	56	18%	None
Speer *et al.*^b^	BNT162b2	18 days	22	18%	46 healthy controls, 43of 46 (93%) had Ab response after 1 dose
Longlune *et al.*^b^	BNT162b2	30 days	HD: 80; PD: 24	HD: 21%; PD: 63%	
Rodriguez-Espinoza *et al.*^b^	mRNA -1273	3 weeks	32 (all PD)	63%	None
Yau *et al.*^b^	BNT162b2	28 days	127	15%	35 health care workers
Second-dose studies (3–4 weeks between doses)					
Attias *et al.*^b^	BNT162b2	3 weeks	52	82%	None
Simon *et al.*	BNT162b2	3 weeks	81	73%	80 healthy controls,median Ab titer significantly lower in HD patients vs. controls
Schrezenmeier *et al.*	BNT162b2	1 and 3 weeks	36	56% 1 week, 89% at 3 weeks	44 nondialysis controls,median Ab titer significantly lower in HD patients vs. controls
Grupper *et al.*	BNT162b2	30 days	56	96%	95 health care workers, median Ab titer significantly lower in HD patients vs. controls
Agur *et al.*	BNT162b2	36 days	HD: 122; PD: 23	HD: 93%; PD: 96%	None
Sattler *et al.*	BNT162b2	8 days	26	85%	39 healthy controls, median Ab titer significantly lower in HD patients vs. controls
Lacson *et al.*	BNT162b2 or mRNA-1273	23 days	186	89%	None
Berar Yanay *et al.*	BNT162b2	21–35 days	160 (127 HD; 33 PD)	90%	132 controls, median Abtiter, significantly lower in HD patients vs. controls
Rincon-Arevalo *et al.*	BNT162b2	3–4 weeks	43	71%	25 healthy controls^a^, median Ab titer significantly in HD patients vs. controls
Frantzen *et al.*	BNT162b2	1 month	212	90%	None
Jahn *et al.*	BNT162b2	2 weeks	72	93%	16 health care workers, median Ab titer significantly lower in HD patients vs. controls
Speer *et al.*^b^	BNT162b2	20 days	22	82%	46 healthy controls, 46of 46 (100%) after 2 doses
Chan *et al.*	mRNA1273	1 week	41	92%	None
Anand *et al.*	BNT162b2, mRNA-1273 or Ad26.COV2S	29 days	519	92%	None
Longlune *et al.*^b^	BNT162b2	30 days	HD: 80; PD: 24	HD: 83%; PD: 85%	None
Rodriguez-Espinoza *et al.*^b^	mRNA-1273	3 weeks	32 (PD)	97%	None
Yau *et al.*^b^	BNT162b2	14 days	127	85%	35 health care workers, all of whom had Ab response after 2 doses
Strengert *et al.*	BNT162b2	21 days	81	95%	34 health care workers, Ab levels significantly lower in HD vs. control

Ab, antibody; COVID-19, coronavirus-2019; HD, hemodialysis; PD, peritoneal dialysis.

a Only data from patients who were COVID-19 naive was extracted wherever presented separately.

b These studies reported antibody response after 1 and 2 doses, and hence appear twice in the table.

**Table 3:** Vaccine response data in transplant population

Study	Vaccine type	Timing after dose	Sample size^a^	Antibody response
One-dose studies				
Boyarsky *et al.*^b^	BNT162b2/mRNA-1273	20 days	322	11%
Benotmane *et al.*^b^	mRNA-1273	28 days	242	11%
Yi *et al.*	BNT162b2/mRNA-1273	28 days	145	6%
Chavarot *et al.*^b^	BNT162b2	28 days	101 (all on belatacept)	2%
Ou *et al.*^b^	BNT162b2/mRNA-1273	22 days	24 SOTRs (23 KTRs); all on belatacept	0%
Two-dose studies (3–4 weeks between doses)				
Marinaki *et al.*	BNT162b2	10 days	34 SOTRs (10 KTRs, 24 heart)	59%
Grupper *et al.*	BNT162b2	16 days	136 SOTRs (125 KTRs)	38%
Benotmane *et al.*^b^	mRNA-1273	28 days	205	48%
Sattler *et al.*	BNT162b2	8 days	39	10%
Husain *et al.*	BNT162b2/mRNA-1273	28 days	28	25%
Rincon-Arevalo *et al.*	BNT162b2	3–4 weeks	40	3%
Boyarsky *et al.*^b^	BNT162b2/mRNA-1273	29 days	322	48%
Rozen-Zvi *et al.*	BNT162b2	28 days	308	36%
Cucchiari *et al.*	mRNA1273	2 weeks	117	30%
Marion *et al.*	BNT126b2^c^	1 month	367 SOTRs	34%
Korth *et al.*	BNT162b2	14 days	23	22%
Chavarot *et al.*^b^	BNT162b2	28 days	101 (all on belatacept)	6%
Ou *et al.*^b^	BNT162b2/mRNA-1273	29 days	24 SOTRs (23 KTRs); all on belatacept	5%

KTR, kidney transplant recipient; SOTR, solid-organ transplant recipient.

a Unless specified, data extracted for kidney transplant recipients.

b These studies reported antibody response after 1 and 2 doses, and hence appear twice in the table. The study by Ou *et al*. assessed a subgroup from the same data set as that used by Boyarsky *et al*., with details on patients who received belatacept.

c Eight of an overall 950 patients in the cohort received mRNA-1273; this analysis includes 367 KTRs, so there were very few to none with mRNA-1273.

### Limitations of Available Studies

Before we review and describe the studies that have reported the response to vaccination in patients with kidney disease, some important limitations should be considered, most of which are common to all currently available studies.

#### Timing

Most studies have reported antibody response very early (the latest is 10 weeks after starting a vaccination course), and antibody response is at its most heterogeneous within these early weeks. In the phase 1 trial of the BNT162b2 mRNA vaccine, the development of anti-S antibodies was delayed by up to 1 week in 65- to 85-year-olds compared with 18- to 55-year-olds.^8^ Similarly, the kinetics described by Attias *et al.* in HD patients seemed to be delayed by approximately 1 week compared with the age-matched Walsh *et al.* cohort.^8^^,^^9^ An analogous delayed response has been reported in solid-organ cancer patients after BNT162b2, given on days 0 and 21, with 38% seropositive at week 3 (*n* = 56), then rising to 95% at week 5 (*n* = 19).^10^ Peak antibody titers should therefore be expected after approximately 4 weeks in patients with significant comorbidities (renal or otherwise). Plotting the renal studies by their percentage seropositivity versus time shows this relationship (Figure 1, with full citations in Tables 2 and 3). Given the difficulties (described in what follows) in comparing these studies directly, we view this as an illustrative framework to interpret the studies rather than as a definitive statistical model.

#### Surrogate Measures of Immunity

Once clinical trials are completed, antibody levels can be used as surrogate measures of vaccine efficacy; however, for the novel coronavirus SARS-CoV-2, there are as yet no internationally accepted standards defining what levels constitute immunity, and assays vary between laboratories.^11^ Therefore, interpretation of antibody levels requires direct experimental comparison with controls who are expected to have immunity---this can be healthy controls similar to those enrolled in the original trials of vaccine efficacy, or convalescent plasma from survivors of COVID-19---and/or evaluation of viral neutralization. The majority of studies in our review did not compare the antibody responses to appropriate controls, used a variety of different assays (10 different assays in the 22 included studies) to detect anti–SARS-CoV-2 antibodies, and did not measure viral neutralization or T-cell immunity, making interpretation of antibody levels difficult. The antibodies measured include IgG, IgA, and/or IgM recombinant spike (anti-S) (monomeric or trimeric, modified to stabilize its conformation), or receptor-binding domain. The latter seem to best correlate with viral neutralization and T-cell responses.^12^ In general, the assays are calibrated by the manufacturer against the wild-type virus circulating in early 2020, but data about cutoffs against emerging variants of interest/concern are not available. Therefore, we have chosen to provide only the proportion of individuals who developed detectable anti–SARS-CoV-2 antibodies (herein called “seropositivity”). Although the World Health Organization initiative to establish a reference sample will go some way to allowing cross-platform comparisons (it has no impact on dynamic ranges), we must not conflate antibody levels reported in these early studies with precise clinical effectiveness.^13^

#### Prior Exposure to SARS-CoV-2

Antibody responses to vaccination should also consider prior immunologic exposure to SARS-CoV-2. For individuals with antigen experience from natural infection, a first vaccination triggers a secondary immune response. As a memory response, this provides antibodies in larger titers and more rapidly than the primary response in individuals without prior exposure. Exposure history is important in both the study (HD or transplant) and comparator populations (often health care workers). Studies that do not measure, or report, baseline serostatus will be liable to comparing primary with secondary responders; this is a particular concern when comparing between studies, where the population prevalence of SARS-CoV-2 may be very different. In a resource-finite setting, the single most informative antibody measure is likely to be >4 weeks after the second dose. Clearly specific research studies may require earlier sampling.

### Chronic Kidney Disease

The effectiveness of vaccination for patients with CKD has been studied at scale as part of the nationwide mass vaccination setting in Israel. The study included 8212 patients with CKD, and reported a 74% protection against subsequent development of severe disease after both doses of BNT162b2 vaccine administration on the 3-week dosing schedule; however, there was a wide confidence interval (−40 to 100), suggesting the findings were based on a small number of events.^14^ Although this was among the lowest rates of protection in the comorbid populations studied, it may represent significant protection from disease for this patient group if confirmed. Other large population-based studies have included large samples of patients with CKD, but specific subgroup analyses have yet to be undertaken.^15^

### Immune-mediated Kidney Diseases

A paucity of data exists on the disease course of COVID-19 in patients on immunosuppression. An analysis of the international registry of COVID infection in glomerulonephritis indicated that mortality rates are comparable to those observed in dialysis and transplant patients.^16^ There are specific considerations in these patient groups, as recently outlined by the Immunonephrology Working Group of the European Renal Association–European Dialysis and Transplant Association.^17^ One concern is the antibody response of patients receiving induction-phase immunosuppression or on maintenance therapy with rituximab, or other B-cell depletion therapies (BCDTs). Studies in rheumatoid arthritis indicate that there was no antibody response to influenza vaccination for 4 to 8 weeks after rituximab administration.^18^ The mRNA vaccine antibody response was studied in a cohort of 133 patients with chronic inflammatory diseases. In that study the use of BCDT was associated with a 36-fold reduction in anti-S IgG and neutralization titers when compared with controls.^19^ Reduced antibody response was more often observed in participants with administration of BCDT within 6 months of vaccination, with a gradual recovery in response 9 months after BCDT. Moreover, glucocorticoid use resulted in a significant decrease in vaccine response, as these patients exhibited a 10-fold reduction in anti-S IgG and neutralization titers.^19^ Another chart review of 89 patients from a rheumatology practice revealed 21 patients (24%) did not develop antibodies after 2 doses of an mRNA vaccine, of whom 20 had been on rituximab and 1 on belimumab.^20^ Similar to the earlier study, none of the 16 patients who received rituximab within 6 months of vaccination developed antibodies and only 1 of the 4 receiving rituximab between 6 and 12 months developed antibodies, whereas 9 of the 10 receiving rituximab ≥12 months before vaccination developed antibodies. From these initial data, the benefit of early vaccine administration after BCDT remains uncertain. Thus, further research is needed to determine the effects on cellular immunity and rate of infections as well as the possible role of additional doses or alternate vaccine strategies in patients with no humoral response to vaccination. A change in strategy is being considered (i.e., use of viral vector–based vaccines) and we hope more informed decisions can be made for this population in the very near future.

With respect to safety, activation of the immune system after vaccination may lead to a relapse or an increase in disease activity, or the declaration of an existing (but undiagnosed) condition. At present, 7 case reports of minimal change disease have been described, 3 of which were relapses that responded to immunosuppression.^21^, ^22^, ^23^, ^24^, ^25^, ^26^ The other 4 patients had a new diagnosis, 3 of them with acute kidney injury. Three of the 4 had responded to immunosuppression in the form of resolution of acute kidney injury, or had a reduction in proteinuria at the time of reporting. Similarly, 9 cases of IgA nephropathy have been described, with 7 known to have IgA nephropathy and in whom there was development of gross hematuria within hours of the vaccine doses, with spontaneous resolution.^27^, ^28^, ^29^, ^30^, ^31^ However, 2 patients were not known to have IgA nephropathy and developed crescentic IgA nephropathy and were still on treatment at the time of reporting. Two patients with cytoplasmic antineutrophil cytoplasmic autoantibody vasculitis have also been reported, as well as 1 patient with a relapse of membranous nephropathy.^31^, ^32^, ^33^ These data are summarized in Table 4. These findings are not unique to COVID vaccination; for example, 4 cases of onset of nephrotic syndrome were identified in the year after meningococcal B vaccination in infants.^34^ In large series, disease relapse after seasonal influenza vaccination is rare, and is probably consistent with underlying background rates.^35^ All but 1 of the cases reported so far occurred after the mRNA vaccine, which may reflect selection bias for the vaccines administered so far. In addition, these represent published cases only, with little information about the total vaccines administered to allow estimates of rates. An ongoing registry will provide more relevant data on how much of these reflect the underlying case rate to be expected or an increase beyond the expected incidence. These cases also emphasize the need for ongoing pharmacovigilance.

**Table 4:** A summary of glomerulonephritis cases and relapses after vaccination

Study	Vaccine	Timing	GN type	Clinical course
Minimal change disease				
Maas *et al.*	BNT162b2	7 days, first dose	New diagnosis, MCD	Steroid responsive
Lebedev *et al.*	BNT162b2	4 days, first dose	New diagnosis, MCD and AKI	AKI resolved, MCD improving with steroids
Agati *et al.*	BNT162b2	7 days, first dose	New diagnosis, MCD and AKI	AKI resolved, still proteinuric at 3 weeks on steroids
Kervella *et al.*	BNT162b2	10 days, second dose	MCD relapse	Steroid responsive
Schwotzer *et al.*	BNT162b2	3 days, first dose	MCD relapse	Responded to steroids + tacrolimus
Holzworth *et al.*	mRNA-1273	1 week, first dose	New diagnosis, MCD and AKI	On treatment
Komaba *et al.*	BNT162b2	8 days, first dose	MCD relapse	Steroid responsive
IgA nephropathy				
Gul Rahim *et al.*	BNT162b2	Hours, second dose	IgA nephropathy, gross hematuria	Spontaneous resolution
Negrea *et al.*	mRNA1273	Hours, second dose	IgA nephropathy, gross hematuria and increased proteinuria	2 patients, spontaneous resolution of hematuria
Perrin *et al.*	mRNA-1273	Second day, first and second dose	IgA nephropathy, gross hematuria	3 patients, 1 with transient proteinuria, spontaneous resolution
RPGN presentations				
Tan *et al.*	BNT162b2	1 day, second dose	IgA nephropathy with fibrocellular crescents, mild IFTA	Underlying IgA, unmasked post vaccination with hematuria
Anderegg *et al.*	mRNA-1273	Second dose	Crescentic IgA nephropathy	Steroid responsive
Sekar *et al.*	mRNA-1273	2 weeks, second dose	Crescentic GN, c-ANCA vasculitis	Dialysis dependent at 2 weeks
Anderegg *et al.*	mRNA-1273	First dose	Crescentic GN, c-ANCA vasculitis	Responded to cyclophosphamide + steroids
Tan *et al*.	BNT 162b2	1 day, second dose	Crescentic GN, anti-GBM	RPGN presentation
Membranous nephropathy				
Aydin *et al.*	Sinovac	2 weeks, first dose	Membranous, relapse	PLA2R positive; remission at 3 months on CNI + steroids, ACEi

ACEi, angiotensin-converting enzyme inhibitor; AKI, acute kidney injury; c-ANCA, cytoplasmic antineutrophil cytoplasmic antibodies; CNI, calcineurin inhibitor; IFTA, interstitial fibrosis an tubular atrophy; MCD, minimal change disease; RPGN, rapidly profilerative glomerulonephritis.

### Vaccine Response in Dialysis

The presence of defects in both humoral and cellular immunity are common in this population, which was well known even before the pandemic.^36^ Responses to vaccination to both hepatitis B and influenza are known to be suboptimal.^37^ Responders to hepatitis B vaccination are generally younger, have a higher dialysis efficacy and serum albumin, and are less likely to have a diagnosis of diabetes mellitus.^38^ Hence, most public health agencies have appropriately prioritized patients on dialysis based on the high COVID-19 case fatality rate, and inability to self-isolate due to the need to come to the center for dialysis treatments in most instances. However, the efficacy of vaccination in this population remains uncertain because these patients were not included in the original efficacy trials.^39^^,^^40^

Several small studies investigating the role of the COVID-19 vaccine in dialysis patients have been reported recently and are summarized in Table 2.^9^^,^^41^, ^42^, ^43^, ^44^, ^45^, ^46^, ^47^, ^48^, ^49^, ^50^, ^51^, ^52^, ^53^, ^54^, ^55^, ^56^, ^57^, ^58^, ^59^, ^60^, ^61^ The development of anti–SARS-CoV-2 antibodies is variable and depends on several factors, including the time from vaccine administration to antibody measurement and type of antibody assay used. The first dose of an mRNA vaccine does not seem to induce anti–SARS-CoV-2 antibodies in the dialysis population, with the proportion of patients having detectable antibodies ranging from 18% to 43% at 3 to 4 weeks after the first dose, and 1 study reporting a higher response (73%).^9^^,^^43^^,^^49^^,^^62^ Another study also reported higher antibody development (87%), but only a select small group of 31 transplant waitlisted patients were included.^58^ The pooled estimate of the antibody response rate was 45% (95% confidence interval [CI], 32%–58%). Most studies have reported data at 2 to 4 weeks after the first dose, and it is possible that development of antibodies is delayed in dialysis patients. However, in 1 study, patients without detectable anti–SARS-CoV-2 antibodies at 4 weeks did not develop antibodies even after 8 weeks of observation, arguing against a delayed response in this population.^55^ The development of antibodies to 2 doses of the mRNA vaccines was found to be higher, ranging from 68% to 96%, with the caveat of additional time from the doses. The pooled estimate after 2 doses is 89% (95% CI, 85%–91%). A center in France offered a third dose to 12 patients after a nonresponse to 2 doses of BNT162b2. Of these patients, 5 (41.7%) had seroconversion after the third dose.^53^ Limited data exist on the efficacy of viral vector–based vaccines in dialysis patients. One study reported on the outcomes of 17 patients who received a single dose of AZD1222, showing a 71% antibody response compared with 80% with BNT162b2 (*P* = 0.3).^43^ Three studies reported on antibody response in peritoneal dislysis patients, which seemed numerically higher at 63% after 1 dose and 96% to 97% after 2 doses.^46^^,^^52^^,^^53^

In the absence of an internationally accepted surrogate measure of efficacy for SARS-CoV-2 vaccination, it is difficult to know how to interpret antibody levels in dialysis patients. In the studies that compared antibody levels between dialysis and healthy controls (a population expected to have “optimal” antibody response), antibody levels in dialysis patients were significantly lower.^41^^,^^42^^,^^44^^,^^45^^,^^47^^,^^51^^,^^56^^,^^62^ Older age and immunosuppression or chemotherapy were associated with lower antibody levels and nonresponse in most studies, whereas other variables associated with no or reduced response included lower serum albumin, lower dialysis vintage, higher comorbidity, and higher intravenous iron sucrose doses in some studies. With respect to safety, fewer side effects were reported after BNT162b2 administration in dialysis patients.^56^

A few studies reported SARS-CoV-2 infections after vaccination. Among the 6 reported infections (from a cohort of 127 HD patients at ≥7 days after the second dose), a 79-year-old male patient developed a severe disease course.^42^ These patients all belonged to the group with the lowest antibody quartile. In a study by Goupil *et al.*, 3 patients developed severe disease with COVID-19, with 1 death and another in intensive care, but after the first dose of vaccine only.^55^

### Kidney Transplant Recipients

The antibody response in kidney transplant recipients to hepatitis vaccines is comparable to that seen in the dialysis population.^63^, ^64^ In contrast, the antibody response to mRNA vaccines in this population has been reported to be poor in several studies, ranging from 0% to 17% after the first dose and from 3% to 59% after 2 doses of mRNA vaccine (Table 3).^44^^,^^47^^,^^58^^,^^64^, ^65^, ^66^, ^67^, ^68^, ^69^, ^70^, ^71^, ^72^, ^73^, ^74^, ^75^, ^76^ The pooled estimate of antibody response after the first dose was 8% (95% CI, 5%–15%) and after the second dose was 35% (95% CI, 29%–42%).^66^ Sattler *et al.* reported detectable spike-specific CD8^+^ T cells in only 2 of 39 kidney transplant recipients and a strongly impaired interleukin-2 production, whereas responder rates for CD4^+^ T helper cells were comparable to those of dialysis patients and controls.^47^ Further analyses indicated that, among kidney transplant recipients, the number of antigen-specific B cells was lower, and these patients also exhibited signatures of inappropriate B-cell memory induction.^44^ So far, 5 studies have reported antibody levels with a control group for comparison and consistently reported a diminished response among transplant recipients.^44^^,^^47^^,^^67^^,^^68^^,^^76^ Consistent risk factors for a lack of antibody response were older age, less time since transplant, and higher immunosuppression (maintenance with antimetabolites, belatacept, triple immunosuppression).^64^, ^65^, ^66^, ^67^ Two studies included only patients on belatacept, with extremely low levels of antibody response (0% and 2%) after the first vaccine dose, which did not increase much after the second dose (5% and 6%).^66^^,^^73^ Similarly, another study reported that none of the 6 patients on belatacept developed antibodies after 2 doses.^71^ Yet another study reported a superior antibody response to mRNA-1273 (60%) over BNT162b2 (49%), and we look forward to seeing whether this finding remains consistent across other studies.^72^ Notably, all studies reported so far have involved mRNA vaccines, and no information is currently available on the immune response to other vaccine platforms for transplant recipients.

Apart from antibody response, several case series reported development of COVID-19 after vaccination.^68^^,^^77^, ^78^, ^79^, ^80^, ^81^, ^82^, ^83^ In the study by Grupper *et al.*, 2 patients (of 136) developed COVID-19 after full vaccination.^68^ In the study by Rozen-Tvi *et al.*, 4 patients (of 308) developed COVID-19 after full vaccination, including 1 with mild disease and 3 with severe disease, of whom 1 died.^71^ Another case series reported on 7 solid-organ transplant recipients who developed COVID-19 despite 1 dose (*n* = 2) or 2 doses (*n* = 5) of mRNA vaccine.^79^ These recipients presenting with COVID-19 had undetectable or low titers of antibodies at the time of infection, and the disease course was similar to that of nonvaccinated patients.^79^ However, in another case series, 13 patients developed COVID-19 (8 after 2 doses and the others after 1 dose of the 3 vaccines), of whom 3 were hospitalized, 2 were in the intensive care unit, and 1 died.^78^ A case series from India, reported 4 cases after immunization with the adenovirus vector vaccine (AZD1222) of whom 2 developed after 1 dose and 2 after 2 doses. As of this writing, 1 of these patients died, 2 were on mechanical ventilation, and 1 recovered.^83^ The largest case series so far is from France where 55 patients developed COVID-19; 11 patients required hospitalization, 6 required intensive care, and 3 died. In that study antibodies were measured in 25 patients, 24 of whom had no response and 1 had a weak titer.^77^ Overall, however, we do not have a good sense of the actual rate of vaccine breakthrough, but it does not seem uncommon and is associated with an absent antibody response or lower antibody titers.

As of this writing, there have been 2 reports of acute rejection after vaccination for COVID-19.^74^^,^^84^ This has raised some alarm on social media, but these reports must be interpreted with caution. There have been reports of low levels of *de novo* anti–human leukocyte antigen antibody development after vaccination for seasonal influenza and H1N1 (swine flu), but they were of uncertain clinical significance.^85^ Although a causal link is difficult to prove on the basis of only 2 case reports so far, postlicensing surveillance will be crucial to monitor and confirm or refute a possible signal. In addition, at this time, transplant patients and health care workers must not delay in having the COVID-19 vaccination based on these sparse data. The current data suggest there may be a diminished antibody response in this population, but maintenance immunosuppression must be maintained throughout the vaccination period and not reduced, as is being advocated in some patients with rheumatologic conditions who are on disease-modifying antirheumatic drugs. The risk–benefit ratio when weighed against acute rejection for patients with a kidney transplant is very different from that of patients with rheumatologic diseases.^86^ Otherwise, the safety data on vaccination in transplant recipients has been very reassuring based on 2 large case series, with mostly local reactions and no anaphylaxis or neurologic reactions.^74^

### Summary and Suggestions

Vaccines are generally well tolerated in patients with kidney diseases. There is a clear recommendation to undergo vaccination. Evidence from early vaccine studies indicates a low antibody response rate after 1 dose in dialysis patients, increasing to a possibly adequate antibody response rate after 2 doses, but antibody levels remain diminished compared with controls. In addition, if antibody levels wane with time, booster doses will likely be needed, and possibly sooner than in the general population. The situation is different in kidney transplant recipients who fail (at least during the time frame of these studies) to mount a detectable humoral response in a majority of patients, and alternative strategies may be needed. It seems essential that close contacts/household members of transplant recipients are prioritized to provide some form of protection. Early studies have highlighted that the density of immunosuppression correlates with vaccine response and, specifically, antimetabolites impair antibody response. It is of importance to fully vaccinate (i.e., with 2 doses) dialysis patients on the deceased donor transplant waitlist, or those about to undergo live donor kidney transplantation, as their posttransplant response will be more severely impaired when compared with the response while still on dialysis.

Reduction of vaccine hesitancy seems pivotal, and some centers may adopt a policy of denial of kidney transplantation if patients refuse vaccination for nonmedical reasons.^87^ Several arguments may reinforce such a strategy, but ethical considerations must be considered and withdrawal from the waitlist may be associated with legal issues. A national survey with 1515 responders from the US indicated about 20% vaccine hesitancy, driven by concerns of adverse effects.^88^ Black, Native American, and younger age women have shown greater vaccine hesitancy. Vaccine hesitancy among staff needs to be reduced, and experience from New York City indicates that thorough programs can reduce hesitancy to a low percentage.^89^

A systematic assessment of new-onset disease or future relapse of glomerular diseases and acute rejection episodes in kidney transplant recipients is an important part of the ongoing pharmacovigilance. Similar innate immune pathways are induced by mRNA vaccines and in systemic lupus erythematosus.^5^ A monitoring of self-reported symptoms should be systematically collected during follow-up, as is being done with in VACOLUP study.^90^

As discussed earlier, patients with BCDT show an impaired humoral response to COVID-19 vaccine. Several recommendations have been made focusing on these patients. The American College of Rheumatology suggests vaccination 4 weeks before the next cycle and administering the next rituximab dose 2 to 4 weeks after the second vaccine dose, if disease activity allows.^91^ A delay in treatment should not increase relapse risk of the underlying kidney disease. The European League Against Rheumatism and the Immunonephrology Working Group of European Renal Association–European Dialysis and Transplant Association recommend that vaccination should be performed 6 months after the last rituximab dose.^17^^,^^91^ Even this approach may not be sufficient and a potential further approach is the switch to alternative immunosuppressive measures, that is, azathioprine in the maintenance of cytoplasmic antineutrophil cytoplasmic autoantibody–associated vasculitis, to increase the likelihood of achieving a humoral response. Studies are forthcoming on the adoption of center-based changes in the management of immune-mediated kidney diseases.

### Where Do We Go From Here?

*Large studies with standardized antibody platforms:* Studies are needed to report data from several centers within one country or, for instance, the European Union. The REnal patients COVID-19 VACcination study is one such ongoing effort.^92^ The studies discussed in this review sketch a landscape; the detailed shading needs directly comparable data at scale.

*Role of routine clinical testing of vaccine response:* The renal community routinely monitors antibody response to viruses (e.g., for hepatitis B in dialysis patients), and, in the case of COVID-19, this may allow for better management strategies.

*Understanding the low immune response in transplant recipients:* Further investigations are needed to understand the low ability of kidney transplant recipients to elicit immunogenicity toward COVID-19 vaccines, as well as test different interventional responses to improve immunogenicity, such as additional booster doses, or using different vaccine platforms.

*Investigations of COVID-19 severity in vaccine nonresponders:* So far, it is unclear whether patients with no antibody response after vaccination are protected or not from severe disease courses. Antibody is only a facet of the vaccine response and other components of the response (e.g., primed T lymphocytes) may compensate to some degree.

*Ideal strategies to boost the immune system:* As more vaccine platforms become available, it may be of interest to switch vaccine platforms in nonresponders (i.e., viral vector–based vaccines after failure to achieve a response after mRNA vaccines) or the role of additional booster doses.

*Defining ideal assays to measure antibody response and perform studies on comparability:* There is a particular need to define threshold titers that can accurately predict clinical protection from mild, moderate, and severe disease caused by the prevailing variant in the community.

*Serial measurement of antibody levels:* As patients have a weaker response compared with healthy individuals, antibody levels should be studied during follow-up; respective information needs to be collected to provide information about ideal timelines. This would also allow for planning booster doses if necessary.

*Specific situations (i.e., response in glomerulonephritis patients):* It is necessary to understand the impact of commonly used immunosuppressive measures, such as steroids and rituximab, the impact of vaccination on relapse risk, and the factors associated with reduced or absent vaccine response---both in terms of antibodies and cellular activity.

In conclusion, the early data that describe the antibody response after vaccination against COVID suggest the response may be lower in patients with CKD compared with the general population; however (particularly for patients with CKD and those on dialysis), there are reasons to be optimistic that the response is robust for many. As more data are published, they will be continually updated on the NephJC COVID-19 vaccine page (http://www.nephjc.com/news/covid-vaccine). The response appears diminished for patients with a kidney transplant and those on immunosuppressive therapy, but again there is evidence of vaccines having a measurable effect. Larger data sets that measure antibody neutralization and outcome data after vaccination in all these patient groups are required to definitively establish vaccine effectiveness in patients with kidney disease and we strongly encourage international collaboration and data sharing between research groups toward this end.

## Disclosures

GA is an employee of Satellite Healthcare and a consultant for Akebia (outside the submitted work). AK has received personal fees from Novartis, Terumo BCT, Miltenyi Biotech, Vifor Pharma, and Alexion (outside the submitted work). JT has received personal fees from Cara Therapeutics, Bayer, Tricida, and AstraZeneca (outside the submitted work). The remaining authors declared no competing interests.

## Acknowledgments

EJC was supported by the 10.13039/100010438Francis Crick Institute which receives its core funding from 10.13039/501100000289Cancer Research UK (FC001827), the UK 10.13039/501100000265Medical Research Council (FC001827), and the 10.13039/100010269Wellcome Trust (FC001827). This research was funded in whole, or in part, by the 10.13039/100010269Wellcome Trust (FC001827). For the purpose of Open Access, the author has applied a CC BY public copyright licence to any Author Accepted Manuscript version arising from this submission. SH receives research salary support from the Department of Medicine, University of Ottawa.

## References

[1] Williamson E.J., Walker A.J., Bhaskaran K. (2020). Factors associated with COVID-19-related death using OpenSAFELY. Nature.

[2] Francis A., Baigent C., Ikizler T.A., Cockwell P., Jha V. (2021). The urgent need to vaccinate dialysis patients against severe acute respiratory syndrome coronavirus 2: a call to action. Kidney Int.

[3] Major R., Selvaskandan H., Makkeyah Y.M. (2020). The exclusion of patients with CKD in prospectively registered interventional trials for COVID-19---a rapid review of international registry data. J Am Soc Nephrol.

[4] Glenn D.A., Hegde A., Kotzen E. (2021). Systematic review of safety and efficacy of COVID-19 vaccines in patients with kidney disease. Kidney Int Rep.

[5] Windpessl M., Bruchfeld A., Anders H.J. (2021). COVID-19 vaccines and kidney disease. Nat Rev Nephrol.

[6] Krueger K.M., Ison M.G., Ghossein C. (2020). Practical guide to vaccination in all stages of CKD, including patients treated by dialysis or kidney transplantation. Am J Kidney Dis.

[7] DerSimonian R., Laird N. (1986). Meta-analysis in clinical trials. Control Clin Trials.

[8] Walsh E.E., Frenck R.W., Falsey A.R. (2020). Safety and immunogenicity of two RNA-based Covid-19 vaccine candidates. N Engl J Med.

[9] Attias P., Sakhi H., Rieu P. (2021). Antibody response to the BNT162b2 vaccine in maintenance hemodialysis patients. Kidney Int.

[10] Monin L., Laing A.G., Muñoz-Ruiz M. (2021). Safety and immunogenicity of one versus two doses of the COVID-19 vaccine BNT162b2 for patients with cancer: interim analysis of a prospective observational study. Lancet Oncol.

[11] Galipeau Y., Greig M., Liu G., Driedger M., Langlois M.A. (2020). Humoral responses and serological assays in SARS-CoV-2 infections. Front Immunol.

[12] Rogers T.F., Zhao F., Huang D. (2020). Isolation of potent SARS-CoV-2 neutralizing antibodies and protection from disease in a small animal model. Science.

[13] (2021). WHO international standard for anti-SARS-CoV-2 immunoglobulin. Lancet.

[14] Barda N., Dagan N., Balicer R.D. (2021). BNT162b2 mRNA Covid-19 vaccine in a nationwide mass vaccination setting. Reply. *N Engl J Med*.

[15] Vasileiou E., Simpson C.R., Shi T. (2021). Interim findings from first-dose mass COVID-19 vaccination roll-out and COVID-19 hospital admissions in Scotland: a national prospective cohort study. Lancet.

[16] Waldman M., Soler M.J., García-Carro C. (2021). Results from the IRoc-GN international registry of patients with COVID-19 and glomerular disease suggest close monitoring. Kidney Int.

[17] Kronbichler A., Anders H.J., Fernandez-Juárez G.M. (2021). Recommendations for the use of COVID-19 vaccines in patients with immune-mediated kidney diseases. Nephrol Dial Transplant.

[18] Westra J., van Assen S., Wilting K.R. (2014). Rituximab impairs immunoglobulin (Ig)M and IgG (subclass) responses after influenza vaccination in rheumatoid arthritis patients. Clin Exp Immunol.

[19] Deepak P., Kim W., Paley M.A. (2021). Glucocorticoids and B cell depleting agents substantially impair immunogenicity of mRNA vaccines to SARS-CoV-2. medRxiv.

[20] Spiera R., Jinich S., Jannat-Khah D. (2021). Rituximab, but not other antirheumatic therapies, is associated with impaired serological response to SARS-CoV-2 vaccination in patients with rheumatic diseases. Ann Rheum Dis.

[21] Schwotzer N., Kissling S., Fakhouri F. (2021). Letter regarding “Minimal change disease relapse following SARS-CoV-2 mRNA vaccine.”. Kidney Int.

[22] Holzworth A., Couchot P., Cruz-Knight W., Brucculeri M. (2021). Minimal change disease following the Moderna mRNA-1273 SARS-CoV-2 vaccine. Kidney Int.

[23] Komaba H., Wada T., Fukagawa M. (2021). Relapse of minimal change disease following the Pfizer-BioNTech COVID-19 vaccine. Am J Kidney Dis.

[24] Maas R.J., Gianotten S., van der Meijden W.A.G. (2021). An additional case of minimal change disease following the Pfizer-BioNTech COVID-19 vaccine. Am J Kidney Dis.

[25] Lebedev L., Sapojnikov M., Wechsler A. (2021). Minimal change disease following the Pfizer-BioNTech COVID-19 vaccine. Am J Kidney Dis.

[26] Kervella D., Jacquemont L., Chapelet-Debout A., Deltombe C., Ville S. (2021). Minimal change disease relapse following SARS-CoV-2 mRNA vaccine. Kidney Int.

[27] Perrin P., Bassand X., Benotmane I., Bouvier N. (2021). Gross hematuria following SARS-CoV-2 vaccination in patients with IgA nephropathy. Kidney Int.

[28] Rahim S.E.G., Lin J.T., Wang J.C. (2021). A case of gross hematuria and IgA nephropathy flare-up following SARS-CoV-2 vaccination. Kidney Int.

[29] Negrea L., Rovin B.H. (2021). Gross hematuria following vaccination for severe acute respiratory syndrome coronavirus 2 in 2 patients with IgA nephropathy. Kidney Int.

[30] Tan H.Z., Tan R.Y., Jun Choo J.C. (2021). Is COVID-19 vaccination unmasking glomerulonephritis?. Kidney Int.

[31] Anderegg M.A., Liu M., Saganas C. (2021). De novo vasculitis after mRNA-1273 (Moderna) vaccination. Kidney Int.

[32] Aydin M.F., Yildiz A., Oruc A. (2021). Relapse of primary membranous nephropathy after inactivated SARS-CoV-2 virus vaccination. Kidney Int.

[33] Sekar A., Campbell R., Tabbara J., Rastogi P. (2021). ANCA glomerulonephritis post Moderna COVID-19 vaccination. Kidney Int.

[34] De Serres G., Billard M.N., Gariépy M.C. (2019). Nephrotic syndrome following four-component meningococcal B vaccination: epidemiologic investigation of a surveillance signal. Vaccine.

[35] Kostianovsky A., Charles P., Alves J.F. (2012). Immunogenicity and safety of seasonal and 2009 pandemic A/H1N1 influenza vaccines for patients with autoimmune diseases: a prospective, monocentre trial on 199 patients. Clin Exp Rheumatol.

[36] Cohen G. (2020). Immune dysfunction in uremia 2020. Toxins.

[37] Zimmermann P., Curtis N. (2019). Factors that influence the immune response to vaccination. Clin Microbiol Rev.

[38] Udomkarnjananun S., Takkavatakarn K., Praditpornsilpa K. (2020). Hepatitis B virus vaccine immune response and mortality in dialysis patients: a meta-analysis. J Nephrol.

[39] Polack F.P., Thomas S.J., Kitchin N. (2020). Safety and efficacy of the BNT162b2 mRNA Covid-19 vaccine. N Engl J Med.

[40] Baden L.R., El Sahly H.M., Essink B. (2021). Efficacy and safety of the mRNA-1273 SARS-CoV-2 vaccine. N Engl J Med.

[41] Grupper A., Sharon N., Finn T. (2021). Humoral response to the Pfizer BNT162b2 Vaccine in patients undergoing maintenance hemodialysis. Clin J Am Soc Nephrol.

[42] Yanay N.B., Freiman S., Shapira M. (2021). Experience with SARS-CoV-2 BNT162b2 mRNA vaccine in dialysis patients. Kidney Int.

[43] Billany R.E., Selvaskandan H., Adenwalla S.F. (2021). Seroprevalence of antibody to S1 spike protein following vaccination against COVID-19 in patients receiving hemodialysis: a call to arms. Kidney Int.

[44] Rincon-Arevalo H., Choi M., Stefanski A.-L. (2021). Impaired antigen-specific memory B cell and plasma cell responses including lack of specific IgG upon SARS-CoV-2 BNT162b2 vaccination among kidney transplant and dialysis patients. medRxiv.

[45] Schrezenmeier E., Bergfeld L., Hillus D. (2021). Immunogenicity of COVID-19 Tozinameran vaccination in patients on chronic dialysis. medRxiv.

[46] Agur T., Ben-Dor N., Goldman S. (2021). Antibody response to mRNA SARS-CoV-2 vaccine among dialysis patients---a prospective cohort study. Nephrol Dial Transplant.

[47] Sattler A., Schrezenmeier E., Weber U. (2021). Impaired humoral and cellular immunity after SARS-CoV2 BNT162b2 (Tozinameran) prime-boost vaccination in kidney transplant recipients. medRxiv.

[48] Lacson E., Argyropoulos C.P., Manley H.J. (2021). Immunogenicity of SARS-CoV-2 vaccine in dialysis. medRxiv.

[49] Torregiani M., Blanchi S., Fois A., Fessi H., Piccoli G.B. (2021). Neutralizing SARS-CoV-2 antibody response in dialysis patients after the first dose of the BNT162b2 mRNA COVID-19 vaccine: the war is far from being won. Kidney Int.

[50] Frantzen L., Cavaille G., Thibeaut S., El-Haik Y. (2021). Efficacy of the BNT162b2 mRNA Covid-19 vaccine in a hemodialysis cohort. Nephrol Dial Transplant.

[51] Jahn M., Korth J., Dorsch O. (2021). Humoral response to SARS-CoV-2-vaccination with BNT162b2 (Pfizer-BioNTech) in patients on hemodialysis. Vaccines.

[52] Rodríguez-Espinosa D., Broseta J.J., Maduell F., Bedini J.L., Vera M. (2021). Humoral response of mRNA-1273 SARS-CoV-2 vaccine in peritoneal dialysis patients. Kidney Int.

[53] Longlune N., Nogier M.B., Miedougé M. (2021). High immunogenicity of a messenger RNA based vaccine against SARS-CoV-2 in chronic dialysis patients. Nephrol Dial Transplant.

[54] Speer C., Göth D., Benning L. (2021). Early humoral responses of hemodialysis patients after COVID-19 vaccination with BNT162b2. Clin J Am Soc Nephrol.

[55] Goupil R., Benlarbi M., Beaubien-Souligny W. (2021). Short-term antibody response after 1 dose of BNT162b2 vaccine in patients receiving hemodialysis. CMAJ.

[56] Simon B., Rubey H., Treipl A. (2021). Haemodialysis patients show a highly diminished antibody response after COVID-19 mRNA vaccination compared to healthy controls. Nephrol Dial Transplant.

[57] Anand S., Montez-Rath M.E., Han J. (2021). Antibody response to COVID-19 vaccination in patients receiving dialysis. medRxiv.

[58] Yi S.G., Knight R.J., Graviss E.A. (2021). Kidney transplant recipients rarely show an early antibody response following the first COVID-19 vaccine administration. Transplantation.

[59] Chan L., Fuca N., Zeldis E., Campbell K., Shaikh A. (2021). Antibody response to mRNA-1273 SARS-CoV-2 vaccine in hemodialysis patients with and without prior COVID-19. Clin J Am Soc Nephrol.

[60] Yau K, Abe KT, Naimark D, et al. The humoral response to the BNT162b2 vaccine in hemodialysis patients 2021. 10.1101/2021.05.24.21257425

[61] Strengert M, Becker M, Ramos GM, et al. Cellular and humoral immunogenicity of a SARS-CoV-2 mRNA vaccine in patients on hemodialysis. 2021. 10.1101/2021.05.26.21257860 PMC8357427 34391096

[62] Goupil R., Benlarbi M., Beaubien-Souligny W. (2021). Short-term antibody response and tolerability after one dose of BNT162b2 vaccine in patients receiving hemodialysis: a report from the Quebec Renal Network COVID-19 study. medRxiv.

[63] Prasoppokakorn T., Vanichanan J., Chaiteerakij R. (2021). A randomized controlled trial of comparative effectiveness between the 2 dose and 3 dose regimens of hepatitis a vaccine in kidney transplant recipients. Sci Rep.

[64] Boyarsky B.J., Werbel W.A., Avery R.K. (2021). Immunogenicity of a single dose of SARS-CoV-2 messenger RNA vaccine in solid organ transplant recipients. JAMA.

[65] Benotmane I., Gautier-Vargas G., Cognard N. (2021). Low immunization rates among kidney transplant recipients who received 2 doses of the mRNA-1273 SARS-CoV-2 vaccine. Kidney Int.

[66] Chavarot N., Ouedrani A., Marion O. (2021). Poor Anti-SARS-CoV-2 humoral and T-cell responses after 2 injections of mRNA vaccine in kidney transplant recipients treated with belatacept. Transplantation.

[67] Marinaki S., Adamopoulos S., Degiannis D. (2021). Immunogenicity of SARS-CoV-2 BNT162b2 vaccine in solid organ transplant recipients. Am J Transplant.

[68] Grupper A., Rabinowich L., Schwartz D. (2021). Reduced humoral response to mRNA SARS-Cov-2 BNT162b2 vaccine in kidney transplant recipients without prior exposure to the virus. Am J Transplant.

[69] Benotmane I., Gautier-Vargas G., Cognard N. (2021). Weak anti-SARS-CoV-2 antibody response after the first injection of an mRNA COVID-19 vaccine in kidney transplant recipients. Kidney Int.

[70] Husain S.A., Tsapepas D., Paget K.F. (2021). Post-vaccine anti-SARS-CoV-2 spike protein antibody development in kidney transplants recipients. Kidney Int Rep.

[71] Rozen-Zvi B., Yahav D., Agur T. (2021). Antibody response to SARS-CoV-2 mRNA vaccine among kidney transplant recipients: a prospective cohort study. Clin Microbiol Infect.

[72] Boyarsky B.J., Werbel W.A., Avery R.K. (2021). Antibody response to 2-dose SARS-CoV-2 mRNA vaccine series in solid organ transplant recipients. JAMA.

[73] Ou M.T., Boyarsky B.J., Chiang T.P.Y. (2021). Immunogenicity and reactogenicity after SARS-CoV-2 mRNA vaccination in kidney transplant recipients taking belatacept. Transplantation.

[74] Cucchiari D., Egri N., Bodro M. (2021). Cellular and humoral response after mRNA-1273 SARS-CoV-2 vaccine in kidney transplant recipients. Am J Transplant.

[75] Marion O., Del Bello A., Abravanel F. (2021). Safety and immunogenicity of anti-SARS-CoV-2 messenger RNA vaccines in recipients of solid organ transplants. Ann Intern Med.

[76] Korth J., Jahn M., Dorsch O. (2021). Impaired humoral response in renal transplant recipients to SARS-CoV-2 vaccination with BNT162b2 (Pfizer-BioNTech). Viruses.

[77] Caillard S., Chavarot N., Bertrand D. (2021). Occurrence of severe COVID-19 in vaccinated transplant patients. Kidney Int.

[78] Tsapepas D., Paget K., Mohan S., Cohen D.J., Husain S.A. (2021). Clinically significant COVID-19 following SARS-CoV-2 vaccination in kidney transplant recipients. Am J Kidney Dis.

[79] Wadei H.M., Gonwa T.A., Leoni J.C., Shah S.Z., Aslam N., Speicher L.L. (2021). COVID-19 infection in solid organ transplant recipients after SARS-CoV-2 vaccination. Am J Transplant.

[80] Song C.C., Christensen J., Kumar D., Vissichelli N., Morales M., Gupta G. (2021). Early experience with SARs-CoV-2 mRNA vaccine breakthrough among kidney transplant recipients. Transpl Infect Dis.

[81] Tau N., Yahav D., Schneider S., Rozen-Zvi B., Abu Sneineh M., Rahamimov R. (2021). Severe consequences of COVID-19 infection among vaccinated kidney transplant recipients. Am J Transplant.

[82] Ali N.M., Alnazari N., Mehta S.A. (2021). Development of COVID-19 infection in transplant recipients after SARS-CoV-2 Vaccination. Transplantation.

[83] Meshram H.S., Kute V.B., Shah N. (2021). Letter to editor: COVID-19 in kidney transplant recipients vaccinated with Oxford-AstraZeneca COVID-19 vaccine (Covishield): a single center experience from India. Transplantation.

[84] Del Bello A., Marion O., Delas A., Congy-Jolivet N., Colombat M., Kamar N. (2021). Acute rejection after anti-SARS-CoV-2 mRNA vaccination in a kidney-transplant patient. Kidney Int.

[85] Katerinis I., Hadaya K., Duquesnoy R. (2011). De novo anti-HLA antibody after pandemic H1N1 and seasonal influenza immunization in kidney transplant recipients. Am J Transplant.

[86] Knoll G.A., MacDonald I., Khan A., Van Walraven C. (2003). Mycophenolate mofetil dose reduction and the risk of acute rejection after renal transplantation. J Am Soc Nephrol.

[87] Kates O.S., Stohs E.J., Pergam S.A. (2021). The limits of refusal: an ethical review of solid organ transplantation and vaccine hesitancy. Am J Transplant.

[88] Garcia P., Montez-Rath M.E., Moore H. (2021). SARS-CoV-2 vaccine acceptability in patients on hemodialysis: a nationwide survey. J Am Soc Nephrol.

[89] Pamplona G.M., Sullivan T., Kotanko P. (2021). COVID-19 vaccination acceptance and hesitancy in dialysis staff: first results from New York City. Kidney Int Rep.

[90] VACOLUP–CRMR des maladies auto-immunes de Strasbourg (RESO) Accessed May 1, 2021. https://maladie-autoimmune.fr/vacolup.

[91] Kant S., Kronbichler A., Salas A., Bruchfeld A., Geetha D. (2021). Timing of COVID-19 vaccine in the setting of anti-CD20 therapy: a primer for nephrologists. Kidney Int Rep.

[92] Kho M.M.L., Reinders M.E.J., Baan C.C. (2021). The RECOVAC IR study: the immune response and safety of the mRNA-1273 COVID-19 vaccine in patients with chronic kidney disease, on dialysis, or living with a kidney transplant---a prospective, controlled, multicenter observational cohort by the REnal patients COVID-19 VACcination (RECOVAC) consortium COVID-19 VACcination (RECOVAC) consortium. Nephrol Dial Transplant.

